# A Gene Circuit Combining the Endogenous I-E Type CRISPR-Cas System and a Light Sensor to Produce Poly-β-Hydroxybutyric Acid Efficiently

**DOI:** 10.3390/bios12080642

**Published:** 2022-08-15

**Authors:** Xiaomeng Li, Wei Jiang, Qingsheng Qi, Quanfeng Liang

**Affiliations:** 1 State Key Laboratory of Microbial Technology, Shandong University, No. 72, Binhai Road, Qingdao 266237, China; 2 The Second Laboratory of Lanzhou Institute of Biological Products Co., Ltd., No. 888, Yanchang Road, Lanzhou 730046, China; 3 Research Center of Basic Medicine, Central Hospital Affiliated to Shandong First Medical University, No. 105, Jiefang Road, Jinan 250013, China

**Keywords:** optogenetics, metabolic engineering, CcaS-CcaR system, type I-E CRISPR system, PHB

## Abstract

‘Metabolic burden,’ which arises when introducing exogenic synthesizing pathways into a host strain, remains a challenging issue in metabolic engineering. Redirecting metabolic flux from cell growth to product synthesis at an appropriate culture timepoint is ideal for resolving this issue. In this report, we introduce optogenetics—which is capable of precise temporal and spatial control—as a genetic switch, accompanied by the endogenous type I-E CRISPRi system in *Escherichia coli* (*E. coli*) to generate a metabolic platform that redirects metabolic flux. Poly-β-hydroxybutyric acid (PHB) production was taken as an example to demonstrate the performance of this platform. A two-to-three-fold increase in PHB content was observed under green light when compared with the production of PHB under red light, confirming the regulatory activity of this platform and its potential to redirect metabolic flux to synthesize target products.

## 1. Introduction

Metabolic engineering is a promising research field that can meet the demands of target compound production while meeting social and environmental obligations [1]. The primary strategy of metabolic engineering is rebuilding and optimizing metabolic networks and regulatory pathways within cells to enhance the production of native metabolites or to enable cells to produce new products, including fuels, chemicals, foods, feeds and pharmaceuticals [2,3]. However, the productivity of desired products is not always ideal, mainly because the native state of engineered strains is influenced by modifications to intrinsic metabolic networks, which causes a ‘metabolic burden’ [4]. Metabolic burden arises because of competition between endogenous and exogenic pathways for substrates and ATP [5]. The downregulation of genes associated with bacterial growth and cell maintenance may increase the titer and yield of target products but, at the same time, decrease the density of engineered bacteria, which may reduce potential productivity levels. Thus, overcoming inefficient production requires resolving the conflict between cell growth and product synthesis. This issue can be remedied by dynamically controlling fermentation during the growth phase, i.e., engineered pathways are inhibited to focus cellular metabolism on biomass build-up and the production phase (in which engineered pathways are induced to biosynthesize the product) [6,7,8].

Several gene expression systems can redirect metabolic flux from mainstream to introduced pathways. Generally, exogenous chemical inducers are combined with soluble transcription factors to achieve the artificial control of gene expression [9], e.g., L-arabinose and isopropyl-beta-D-thiogalactopyranoside (IPTG) for the expression of enzymatic pathways [10,11]. However, the effectiveness of these approaches is limited because of potential toxicity, the high cost of inducers [12], transport process delays [13], off-target effects [14] and incompatibility with industrial scale-up [15]. Moreover, removing residual chemical inducers from the growth medium after induction is difficult, creating challenges for studies that require the precise temporal control of gene expression at the desired levels [16]. Another approach is based on natural metabolite-sensing proteins, where endogenous metabolites act as inducers to trigger the expression of a target gene [17,18]. However, slow responses and a narrow range of sensors limit the general use of this approach [19]. More complex systems like logic gates and toggle switches can also be used to realize self-regulation and complex control [10,11,20,21]. However, difficulties in the management and flexible regulation of complex genetic circuits exist [18,19].

In contrast, the application of optogenetic switches appears to be suitable for precise and complex regulation in various fields. These switches are inexpensive and compatible with any carbon source or nutrient composition [22]. In addition, light can be applied and removed easily, enabling the dynamic and reversible control of genetically engineered circuits [23]. A variety of light sensors exist. For example, the Cph8/OpmR sensor responds to red light [24], the pMag/nMag sensor responds to blue light [25], and the CcaS-CcaR sensor responds to green and red light [26]. Such light sensors provide powerful tools to control cellular processes. Currently, most well-tested light sensors in *E. coli* fall into the categories of phytochromes and the light-oxygen-voltage (LOV) family of proteins. Plant pigments share a photosensitive core, including the PAS (Per-Arnt-Sim) domain; GAF (cGMP-specific phosphodiesterase, adenylate cyclase and FhlA) domain; and PHY (photochrome-specific GAF-associated) domain [27]. The core architecture connects the functional domain of photoreceptors, such as the transmembrane sensor histidine kinase (HK), in two-component systems. Illumination causes the photoisomerization of HK bound to the cofactor, which controls gene expression downstream of the output promoter [21]. LOV domains are members of the PAS domain superfamily and connect with HK and helix-turn-helix (HTH) DNA binding domains through light-induced uncaging, flipping and dimerization to control the fused functional domain [28]. The diversity of light sensors has inspired researchers to conduct in-depth research on their engineering. Optogenetic tools have been used previously for the remote control of metabolic pathways in a bioreactor [29] and in regulating the activity of a specific protein [30]. Previous research constructed an integrated RGB system to respond to multichromatic control and applied this system to control metabolic flux through a metabolic pathway to acetate [31]. Recently, Makoto et al. developed a series of circuits for optogenetic regulation of the lac operon, termed OptoLAC. In this circuit, blue light is used to replace the inducer IPTG to control the engineered metabolic pathway for chemical production. Using OptoLAC increased the production of mevalonate and isobutanol by 24% and 27%, respectively [4]. Evan et al. also constructed a similar optogenetic regulation circuit, OptoAMP. In addition to achieving the light-controlled regulation of gene expression, this circuit also amplifies the transcription response to blue light by as much as 23-fold when compared with the basal circuit, expanding the application of optogenetics in metabolic engineering [32]. The precise and tunable control of light switches can be used to rewire cellular metabolism further.

The optical control gene circuit can be used in metabolic engineering to redistribute metabolic flux to synthesize desired products, but metabolic engineering usually involves complex metabolic pathways, which require the activation or inhibition of multiple genes. Currently, most studies use light-controlled gene circuits in combination with the widely used CRISPR type II system to achieve the redirection of metabolic flux. For example, researchers have proposed an optogenetic CRISPR interference (opto-CRISPRi) technique that allocates metabolic resources based on different optical signal frequencies to allow bacteria to be controlled during growth and production stages [33]. These studies used a blue light-sensitive protein, EL222, to regulate the expression of the dCpf1-mediated CRISPRi system, which closed a competitive pathway and redirected metabolic flux to heterologous muconic acid synthesis in *E. coli* with a 130% increase in production [33]. Furthermore, Polstein et al. designed a light-activated CRISPR-Cas9 effector (LACE) system. This system was accomplished by fusing light-induced heterodimeric proteins CRY2 and CIB1 to the transactivation domain and catalytically inactive *dCas9*, respectively, to induce the transcription of endogenous genes in the presence of blue light [34]. When using the type II CRISPRi system, in addition to designing a specific guide RNA for the target gene, an additional plasmid vector expressing *dCas9* (4 kb) should be constructed. In contrast, the endogenous type I-E CRISPR-Cas system only requires the expression of the CRISPR array and, thus, can be easily used for internal regulation without causing a metabolic burden. Our group applied IPTG-induced type I-E CRISPR-Cas system to the metabolic engineering of *E. coli* for the first time [35].

By taking advantage of the green/red light-sensitive CcaS-CcaR system and the endogenous type I-E CRISPRi system [35], we present a metabolic platform for the precise temporal and spatial redirection of metabolic flux and use poly-β-hydroxybutyric acid (PHB) production as an example to test the platform. This metabolic platform functioned well through fermentation tests using the dry weight of cells and PHB productivity as indicators, thereby supporting our concept. Thus, we illustrate a new strategy in metabolic engineering by fusing optogenetic tools and the endogenous CRISPRi system for precise switching between cell growth and product synthesis.

## 2. Materials and Methods

### 2.1. Construction of Strains and Plasmids

The strains, plasmids and primers used in this work are listed in Supplementary Tables S1–S3. Plasmids without the crRNA sequence were constructed in *E. coli* DH5α cells with the sequence of EE-E15 (lab stock, *E. coli* Top10Δ*cas3*). The strain was attained by knocking out *cas3* and substituting the native promoter of the cascade operon with J23119. CcaS consists of an N-terminal transmembrane helix; a GAF domain, which acts as the sensor domain; a linker region (L1); two PAS domains; a second linker region (L2); and a C-terminal histidine kinase (HK) domain. By removing L1 and the two PAS domains and fusing the GAF and HK domains with the truncated linker region, a truncated version of the photoreceptor can be constructed [36]. The plasmid pSR43.6 was used as a template, and the primers #3 F, #3 R, #4 F, #4 R, #10 F and #10 R were used for PCR amplification. The plasmids pSR43.6#3, pSR43.6#4 and pSR43.6#10 were obtained by Gibson assembly. The above plasmids were transformed into DH5α cells by chemical transformation to obtain strains #3, #4 and #10. The plasmid Paracr15A was used as the plasmid construction template. The primers 58.6 primer F and 58.6 primer R were used for PCR amplification, and the high-copy plasmid Paracr58.6 was obtained by Gibson assembly. The vector plasmids pHZ3.1 and pSR43.6 were PCR-amplified with the crRNA gene fragments, respectively, and the plasmids pHZ-crRNA and 43.6-crRNA were obtained by Gibson assembly. The pHZ-crRNA was used as the plasmid construction template, and the primers phbCAB F2 and phbCAB R were used for PCR amplification. The plasmid vector and the *phbCAB* were assembled by Gibson to obtain the plasmid pHZ-PHBcon. The plasmids Paracr58.6, pHZ-crRNA, 43.6-crRNA and pHZ-PHBcon were transformed into *E. coli* EE-E15 by chemical transformation to obtain the strains Ara58.6, SphZ, S43.6 and SlcrPHB.

### 2.2. Characterization of the CcaS-CcaR System

Bacteria were inoculated in 2 mL of LB culture medium with chloramphenicol and streptomycin in 24-well plates. The plates were placed in a shaker and cultured at 37 °C and 500 rpm for 12 h. The cells were then placed on ice to stop bacteria growth, and 100 μL of the culture was transferred to wells of a 96-well plate to measure OD_600_. The cultures were diluted to an OD_600_ = 0.1 with M9 culture medium (with chloramphenicol and streptomycin) to a final volume of 1 mL. Three parallel repeats were prepared for each sample. The new plates were wrapped with aluminum foil to prevent natural light reaching the cells, and the lighting device was placed in the aluminum foil to ensure that the cells can be illuminated by green/red light. The plate was cultured at 37 °C and 500 rpm. The containers were placed into a microplate reader (Synergy HT, BioTek, Winooski, VT, USA) every hour to measure the cell density and fluorescence. A negative control was used to measure and subtract intrinsic cellular fluorescence.

### 2.3. Characterization of crRNA

Single colonies were inoculated into 50 mL of LB medium with appropriate antibiotics (spectinomycin and chloramphenicol) and cultured at 37 °C and 220 rpm in darkness overnight for 13 h. The samples were diluted to an OD_600_ of 0.06–0.07 using 50 mL of M9 medium (containing 1% glycerol and appropriate antibiotics), and these cultures were placed in Erlenmeyer flasks. Three parallel repeats were prepared for each sample. The cultures in the Erlenmeyer flasks were cultured at 37 °C and 220 rpm under the red-light condition and then changed to green light at 0, 5 and 9 h. Then, 200 μL of the samples were placed into 96-well cell culture clusters using the microplate reader to measure the absorbance every 2 h.

### 2.4. Fermentation of PHB

Single *E. coli* colonies were each inoculated into 6 mL of LB culture medium containing chloramphenicol and streptomycin, and these seeds were cultured for 12 h. Subsequently, 1 mL of the first seed was inoculated into 50 mL of LB culture medium containing chloramphenicol and streptomycin and cultured at 37 °C, 220 rpm for 8 h. Approximately 1 mL of the 50 mL second seed culture was inoculated into 100 mL of M9 culture medium (OD_600_ = 0.05) containing chloramphenicol and streptomycin for fermentation. Glucose was the only carbon resource. Fermentation cultures were grown at 37 °C, 180 rpm for 64 h. The OD and glucose consumption were measured every 8 h. OD was detected with an Ultraviolet-visible spectrophotometer and 15 μL of fermentation culture supernatant was used to analyze glucose consumption in a biosensor analyzer. The PHB content was determined by gas chromatography (GC).

### 2.5. Measurement of PHB Production

After fermentation, the cells were harvested by centrifugation at 7000 rpm for 15 min. The bacteria sample was lyophilized for 6 h, and the cell sample was weighed. The bacteria were ground carefully, and 15 mg of the bacteria sample was accurately weighed into a well-sealed small bottle. The weight of the bottle with the bacteria sample was weighed. Concentrated sulfuric acid (150 μL), 850 μL of methanol and 1 mL of trichloromethane were added in that order under a fume hood. The bottles were sealed and placed into a 100 °C oil bath for 1 h. The bottles were then removed, and 1 mL of ultrapure water was added. The bottles were sealed, and the samples were thoroughly mixed by vortex oscillation. The samples were left standing for at least an hour until the liquid separated. The top water phase and lower organic phase were stored. PHB dissolved into the organic solvent. The organic phase (~600 μL) was carefully transferred to a 1 mL vial for a GC analysis to determine the content of PHB.

The chromatographic column of the gas chromatograph is an Rtx-5 capillary column, and the detector is a hydrogen flame ionization detector (FID). The column temperature was kept at 80 °C for 1 min, then the column temperature was raised to 120 °C at the rate of 10 °C/min, and then to 160 °C at the rate of 45 °C/min. The column temperature was kept for 5 min, with a total time of 10.89 min. The FID temperature was 300 °C, the hydrogen flow rate was 40 mL/min, and the air flow rate was 400 mL/min. The injection port temperature was 250 °C, and helium was used as the carrier for a 1:15 split injection.

## 3. Results and Discussion

### 3.1. Optimization of the Optogenetic CcaS/CcaR Sensor

CcaS-CcaR, a green/red photo-reversible two-component signal transduction system (TCS) first discovered in cyanobacteria [37], is used widely in prokaryotes and eukaryotes. The autophosphorylation of CcaS and downstream phosphorylation of CcaR are upregulated under 672 nm green light irradiation, and then CcaR binds to the cpcG2 promoter region, which initiates the expression of downstream genes. The above process is reversed under red light irradiation.

**Figure 1:**
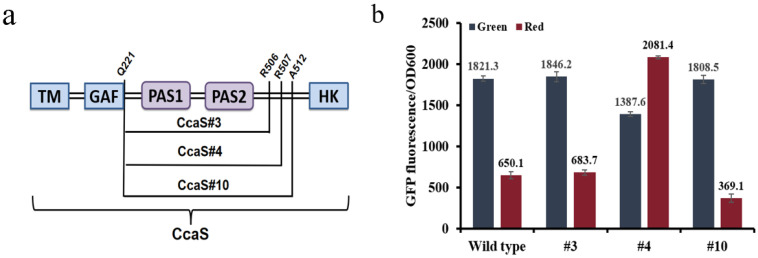
(**a**) Construction of CcaS #3, #4 and #10 variants. (**b**) The fluorescence intensity of cells grown under green light and red light. The fluorescence intensity was acquired 9 h after inoculating the cultures into 24-well plates with a starting OD_600_ of ~0.1. The cultures were exposed to different light to characterize the different expression levels. The basic OD_600_ and fluorescence were eliminated. Bars indicate relative fluorescence intensity when compared with the normalized fluorescence level of the wild-type system. Data represent the mean ± SD from three independent experiments (*n* = 3).

In this study, we expressed the CcaS/CcaR system in *E. coli* TOP10 to explore and improve its regulatory performance. We constructed three variant strains, #3/#4/#10, by removing different lengths of PAS domain and linker DNA [38,39] (Figure 1a), which were co-characterized with the wild-type system as a control. Upon illumination, all sensors exhibited different expression patterns under red and green irradiation (Figure S1). Among them, variant #10 had the highest dynamic ranges of green over red light, whereas the difference between the wild-type and #3 was insignificant. The dynamic ranges of green light over red light for the different systems were 2.8 (wild type); 2.7 (#3); and 4.9 (#10). In contrast, #4 exhibited a reverse response when compared with the other two systems (i.e., #3 and #10), with higher expression under red light instead of green light and an approximate 1.5-fold change (Figure 1b). Thus, we selected the best-performing system, #10, for regulation in the following studies.

**Figure 2:**
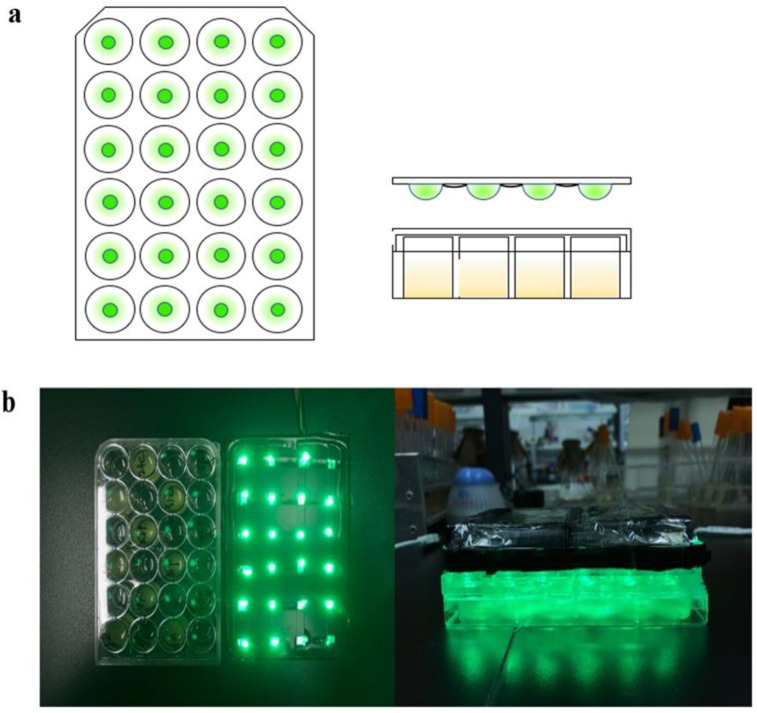
Diagram of the light-emitting diode array of the 24-well plate (LA-WP). (**a**) Design of the LA-WP. Twenty-four SMD LEDs were installed and used to irradiate each well of the 24-well plate separately and independently. (**b**) Photos of the light device.

In optimizing the optical sensor, we developed a 24-well plate read-out combined with an LED patch lamp and optical control hardware to achieve greater penetration of light and an earlier K value of the culture (Figure 2a). The ballast is connected to a 220 V power supply, and 24 LED patch lamps match the 24-well plate (Figure 2b). The light illumination from each patch lamp is controlled independently. The illumination intensity of red light was 5.49 w/m^2^, and that of green light was 3.26 w/m^2^ (Table S4).

### 3.2. Characterization of the Type I-E CRISPRi System

In *E. coli*, the widely used type II system requires an additional plasmid to express dcas9. In contrast, the endogenous type I CRISPR system is relatively easy to regulate internally without causing metabolic burden [35]. CrRNA in the type I-E system sequences is recognized by the ribonucleoprotein complex, Cascade, during target DNA binding [40,41]. The ribonucleoprotein complex Cascade is composed of a 61-nt crRNA and five different Cas proteins in an uneven stoichiometry, which are encoded by eight genes separately (*cas3, cse1, cse2, cas7, cas5, cas6e, cas1, cas2*) [42]. In this system, by knocking out *cas3* and activating the expression of the CRISPR-associated complex for antiviral defense (Cascade), the DNA-cutting function of the CRISPR system is inactivated, whereas the DNA-binding function is maintained, which can inhibit the expression of target genes (Figure S2). After exploring the expression of the single light-controlled gene expression system CcaS/R in *E. coli*, we combined the single light-controlled gene expression system with the endogenous I-E CRISPRi system in *E. coli* to construct a platform for the dynamic regulation of metabolic flux.

**Figure 3:**
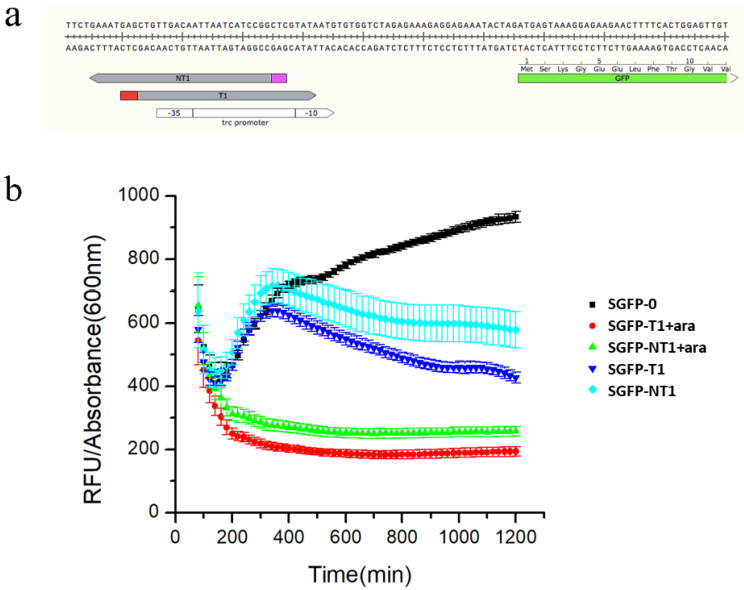
Characterization of the type I−E CRISPRi system (**a**) The site of spacers target *gfp.* (T1 and NT1 target the promoter regions of both strands.) (**b**) Characterization of the type I−E CRISPRi system targeting different sites of *gfp*. Data represent the mean ± SD from three independent repeats (*n* = 3).

To remove the DNA degradation function and maintain the DNA binding function of the modified type I-E CRISPR system, a special *E. coli* TOP10 with the Cas3 protein knocked out, and the promoter of Cascade substituted by the promoter J23119 was constructed and the strain named EE-E15 [35] (Figure S2). In order to verify the function of this system, the GFP-expressing plasmid and crRNA expression vector were co-transformed into *E. coli* TOP10Δ*cas3*, and a series of strains SGFP-Y (Y represents 0, T1 and NT1, which are the spacer names; 0 indicates control, targeting no sites) were constructed to test the function of crRNA targeting different GFP sites (Figure 3a). In the presence of L-arabinose, SGFP-T1 and SGFP-NT1 showed strong inhibition. The results showed that repression among different sites varied widely, and the spacer positioned on the promotor region presented a high repression level (Figure 3b). 

### 3.3. Construction of the Type-I-E CRISPR System for Regulating gltA Expression

**Figure 4:**
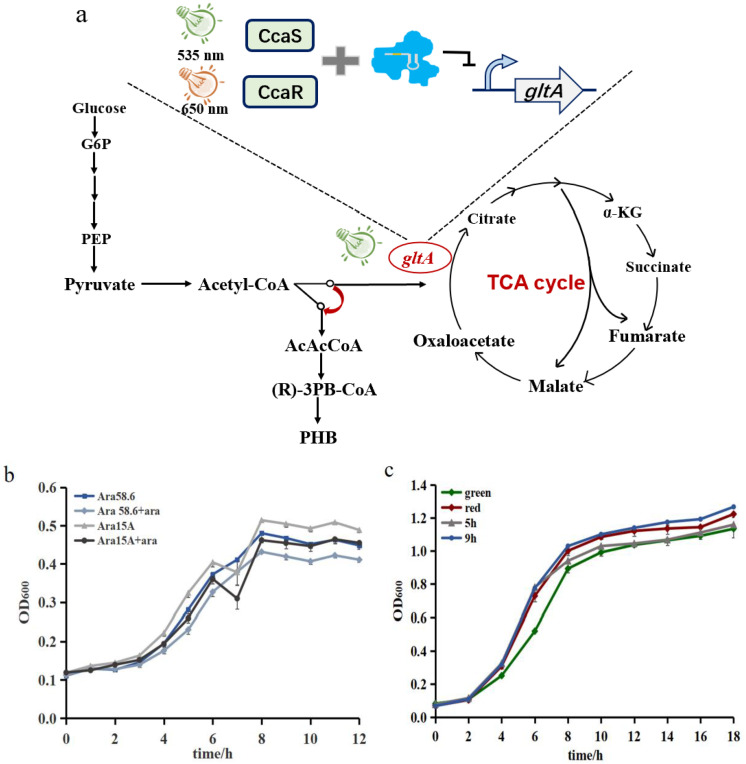
The regulation effect of the endogenous CRISPRi system targeting *gltA*. (**a**) The metabolic pathway to the synthetic product. Two acetyl-CoA will condense to produce acetoacetyl-CoA, which is catalyzed by acetoacetyl-CoA reductase to generate (R)-3-hydroxybutyryl-CoA. Then, it is polymerized by PHA polymerase to form PHB. (**b**) Comparison of the regulation effect between different plasmid copy numbers. Ara58.6 is the strain with the aracr58.6 plasmid, and Ara15A is the strain with the aracr15A plasmid. (**c**) The effect of different regulation times by switching red light to green light. Green and red represent continuous exposure to green and red light, respectively, whereas X h (X = 5.9) represents switching the light at X hours after starting the culture. Data represent the mean ± SD from three independent experiments (*n* = 3).

We focused on citrate synthase as a model candidate for metabolic flux regulation. Citrate synthase catalyzes acetyl-CoA and oxaloacetate to form citric acid, which is the rate-limiting step in the TCA cycle and crucial for bacterial growth [43,44]. Thus, regulating the expression of *gltA*, which codes for citrate synthase, may affect central metabolism. In contrast to previous work using the imported exogenous CRISPRi system (CRISPR/dCas9) [30,45], an endogenous CRISPRi system, type I-E CRISPRi system in *E. coli* was applied to convert the growth phase to the production phase without causing extra metabolic burden and interference. Therefore, we used the type-I-E CRISPR system for regulating *gltA* expression to control metabolic flux (Figure 4a).

In characterizing the type I-E CRISPR system, we constructed strain EE-E15 with *Cas3* knocked out. As demonstrated in other studies, targeting different sites of gene can lead to a different regulation effect for *gltA*, and because the spacer targeting the latter of the two promoters of the *gltA* gene showed the greatest effect [35,46]. Therefore, we selected this promoter as the target site and constructed a medium-copy plasmid (Paracr15A) and a high-copy plasmid (Paracr58.6) containing crRNA targeting *gltA* to inhibit the expression of *gltA* (Figure S3). Initially, we characterized this regulator with chemical substances. Inducing with 0.2% L-arabinose resulted in an apparent suppression of strain growth when compared with the strain grown without L-arabinose (Figure 4b). This observation indicated that the type I-E CRISPRi system affects cell growth by producing crRNA targeting *gltA*. Strains with aracr58.6 showed a slower growth rate, which may be caused by the high leakage of the high-copy plasmid.

We then combined the CcaS #10/CcaR optogenetic sensor with crRNA to investigate the regulation effect of targeting *gltA* through switching light. We constructed the medium-copy plasmid (43.6-crRNA) and high-copy plasmid (pHZ-crRNA) with a specific crRNA spacer and transformed both plasmids into Top10Δ*cas3* cells to gain the strains S43.6 and SphZ, respectively. Upon green or red illumination, the two groups showed variance in growth (Figure S4). The growth of SphZ decreased under green light, and the growth inhibition of S43.6 was not clearly observed, which may be because of the low expression of crRNA and the leakage of CcaS/CcaR under red light. Thus, we selected the high-copy plasmid (pHZ-crRNA) in the subsequent studies.

We then investigated the regulation effects of light-controlled endogenous CRISPRi targeting *gltA* at different induction times by switching red light to green light at 0, 5 and 9 h. The strains exposed to red light grew better than those exposed to green illumination continuously (Figure 4c). Bacteria growth had obvious palliation after switching to the green light at 5 h (K/2 value). In contrast, there was a negligible growth difference between the strains induced at 9 h and the control sample illuminated with red light. Thus, we hypothesize that during the stationary phase, there was effectively no TCA cycle inhibition to repress the cell growth, which may be caused by the metabolic flow of the cells [8].

### 3.4. Demonstrating Light-Controlled Redirection of Metabolic Flux

**Figure 5:**
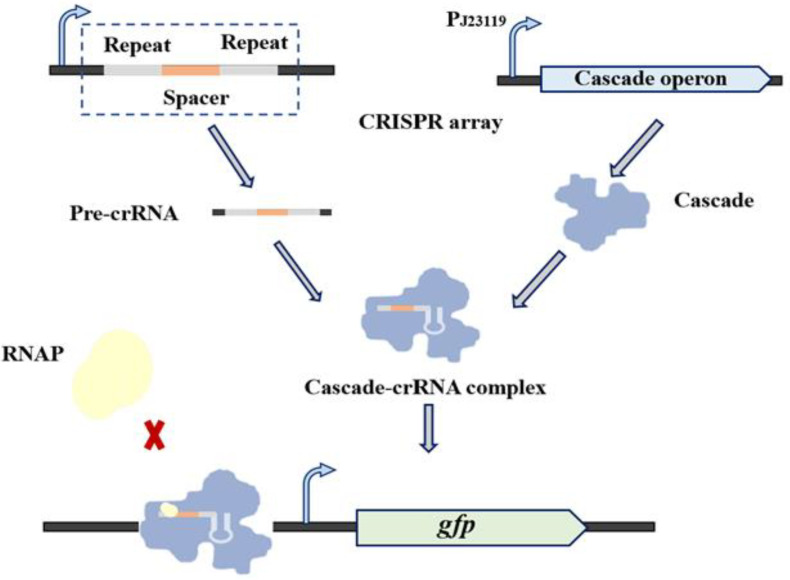
The light-controlled CRISPR system regulates the metabolic pathway of PHB production. The crRNA was expressed on a plasmid and Cascade was activated for expression with the constitutive promoter J23119. This Cascade mediates the maturation of crRNA and forms a complex with crRNA to target gene sites, thus, disturbing transcription.

We used our system to regulate the PHB synthetic pathway as a proof of concept for the practical application of the light-mediated metabolic flux redirection system. The PHB production pathway competes with the TCA cycle for the common substrate acetyl-CoA. Thus, green light was used to activate the I-E CRISPRi system. This system targeted the *gltA* gene, which is the key gene of the TCA cycle. We inhibited the expression of *gltA* at different growth stages to balance the metabolic flux between growth and product production to improve the target product yield. We constructed the strain SlcrPHB, which expressed the phbCAB cluster constitutively and transcript crRNA targeting *gltA* under the control of the CcaS/CcaR system (Figure 5). crRNA was expressed at different growth stages (lag, exponential, stationary phases) during PHB fermentation by switching red to green light irradiation. The groups were name experimental group A (green light irradiation at 0 h inhibition of *gltA*); experimental group B (red light converted to green light irradiation at 5 h inhibition of *gltA*); experimental group C (red light converted to green light irradiation at 13.5 h inhibition of *gltA*); and control group D (red light irradiation, no inhibition of *gltA*). To provide different corresponding lighting for PHB fermentation, the inner wall of each light box was surrounded by a circle of LEDs, and the maximum changes in radiance were related to the number of LEDs.

**Figure 6:**
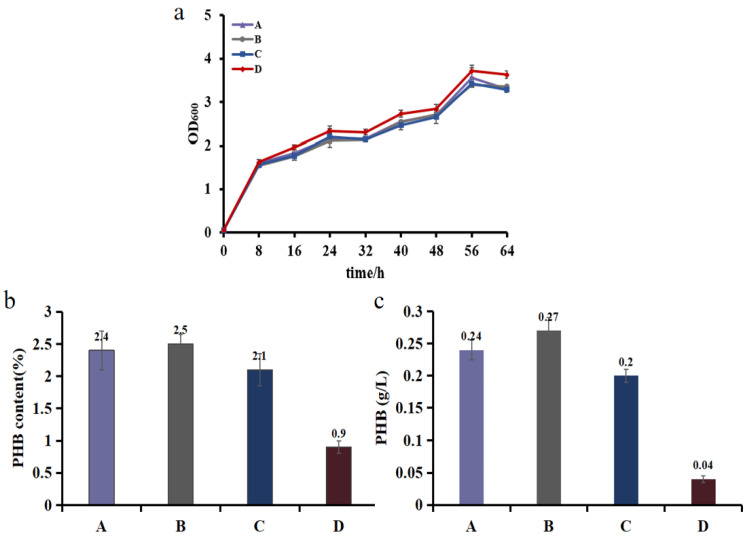
PHB fermentation in various engineered *E. coli* strains. (**a**) Growth of strains. (**b**) PHB content and (**c**) concentration at the end of fermentation. The experiments were performed in duplicate, and error bars indicate standard deviations (SD).

During fermentation, there was no significant difference in the OD_600_ values between SlcrPHB growing under different light conditions (Figure 6a). This observation indicated that the suppression of *gltA* did not affect the growth of *E. coli*, which was probably because PHB had a positive effect on cell growth and offset the detrimental impact of *gltA* knockdown [47].

Moreover, compared with that of control group D under red light irradiation, the PHB contents for experimental group A and experimental group B increased by 2.6- and 2.7-fold, respectively (Figure 6b). This result suggests that inhibiting *gltA* was essential for improving the content of PHB. In contrast, the PHB production of experimental group B was 1.12 and 1.35 times that of experimental group A and experimental group C, respectively, possessing the highest productivity and content (Figure 6c). This result is probably because suppressing *gltA* expression during the lag phase causes detrimental effects on intrinsic metabolism. In contrast, the inhibition of *gltA* during the stationary phase exerts weaker effects on cell growth, whereas a lower level of the carbon source is available for PHB synthesis. This observation indicated that suppressing *gltA* expression during the exponential phase may be a feasible option to optimize PHB production, which is consistent with the work by Soma et al. [8], where later induction resulted in a smaller increase in the concentration of acetate and product accumulation. As fermentation scales, expressing crRNA during the exponential phase may, to some extent, increase profit.

We demonstrated that optogenetic tools are suitable for the metabolic flux regulation of a prokaryotic system. The effect of multi-light channels applied in metabolic engineering to inhibit the expression of acetate-producing genes has been demonstrated previously [30], indicating that introducing optogenetic control in fermentations should increase the yield of target products. We altered light at various time points to accomplish the conditional knockdown of a critical gene to switch the bacterial phase, which distinguishes our work from previous research efforts. Using this approach, bacteria accumulated biomass during the red light-induced growth phase and then, under green light illumination at a particular time point, transformed to the production phase. We can maximize the production of valuable chemicals by regulating the timing of the switch. Moreover, we tuned the expression of citrate synthase to increase the efficiency and universality because we controlled bacterial growth. The knockout or knockdown of citrate synthase has not been used frequently in metabolic engineering because of the adverse effects on cell growth [8]. Previous research showed nearly no *E. coli* growth after knocking out *gltA*, which can be attributed to the inhibition of the TCA cycle [48]. However, endogenous and synthetic pathways compete for the important metabolic precursor, acetyl-CoA. Therefore, the light-controlled conditional inhibition of *gltA* serves as a promising strategy to redirect metabolic flux from the TCA cycle to desired pathways.

## 4. Conclusions

In this report, we demonstrated that a light-controlled gene expression system can be used for the metabolic flux regulation in *E. coli*. After characterizing the effects of different CcaS variants in *E. coli*, a platform that dynamically regulates metabolic flux was constructed by combining the CcaS #10 variant with the endogenous I-E CRISPRi system, and PHB production was taken as an example to illustrate the performance of this metabolic platform. PHB productivity was found to differ by 2–3-fold from green light to red light irradiation, indicating the successful use of the platform. Moreover, we showed that the time point for induction gave different target product yields. Therefore, inhibiting *gltA* at three different time points, the lag phase, logarithmic phase and stationary phase, can significantly improve the effectiveness and universality of light-controlled CRISPR switches. We demonstrated the possibility of combining light-controlled elements with the endogenous I-E CRISPRi system for dynamic regulation studies, providing new research ideas and tools for the precise allocation of metabolic flux.

## Data Availability

Not applicable.

## Supplementary Materials

The following supporting information can be downloaded at: https://www.mdpi.com/article/10.3390/bios12080642/s1, Table S1: Strains used in this study; Table S2: Plasmids used in this study; Table S3: Primers used in this study; Table S4: Parameters of LED strips; Figure S1: The relative fluorescence of different type of CcaS-CcaR; Figure S2: The modification of endogenous CRISPRi system; Figure S3. sgRNA sequence targeting *gltA*; Figure S4: The regulation effect of different copy-number in light-controlled system.
