# Supplementary material for: A Gene Circuit Combining the Endogenous I-E Type CRISPR-Cas System and a Light Sensor to Produce Poly-β-Hydroxybutyric Acid Efficiently
Source: Biosensors (Basel). 2022 Aug 15;12(8):642. doi: 10.3390/bios12080642 (PMC9405541; doi:10.3390/bios12080642)
Supplement: Supplementary file 1 [file biosensors-12-00642-s001.zip › biosensors-1834143-supplementary.pdf]

Supplementary Material

A Gene Circuit Combining the Endogenous I-E Type CRISPR-Cas System and a Light Sensor to Produce Poly-β-Hydroxybutyric Acid Efficiently

Xiaomeng Li [1,2], Wei Jiang [3], Qingsheng Qi [1] and Quanfeng Liang [1,*]

1 State Key Laboratory of Microbial Technology, Shandong University, No. 72, Binhai Road, Qingdao 266237, China;
2 The Second Laboratory of Lanzhou Institute of Biological Products Co., Ltd., No. 888, Yanchang Road, Lanzhou 730046, China
3 Research Center of Basic Medicine, Central Hospital Affiliated to Shandong First Medical University, No. 105, Jiefang Road, Jinan 250013, China
* Correspondence: liangquanfeng@sdu.edu.cn; Tel.: +86-13573163779

Table S1. Strains used in this study

Strains	Description	Source
E.coli TOP10	F- *mcrA* Δ(*mrr-hsdRMS-mcrBC*) *φ80lacZ*Δ*M15* Δ*lacX74 nupG recA*1 *araD139* Δ(*ara-leu)*7697 *galE15 galK16 rpsL(StrR) endA*1 λ-	Lab Stock
E. coli DH5α	F-*end*A1glnV44thi-1*rec*A1*rel*A1*gyr*A96*deoRnup*GΦ80 *lac*ZΔM15Δ(*lac*ZYA-*arg*F)U169，hsdR17(rK-mK+)	Lab Stock
EE-E15	*E. coli* TOP10(Δ*cas3*)	Lab Stock
Wide type	DH5α carrying pHZ3.1 and pSR43.6	This study
#3	DH5α carrying pHZ3.1 and pSR43.6#3	This study
#4	DH5α carrying pHZ3.1 and pSR43.6#4	This study
#10	DH5α carrying pHZ3.1 and pSR43.6#10	This study
SphZ	EE-E15 carrying pHZ-crRNA	This study
S43.6	EE-E15 carrying 43.6-crRNA	This study
Ara58.6	EE-E15 carrying Paracr58.6	This study
Ara15A	EE-E15 carrying Paracr15A	Lab Stock
SlcrPHB	EE-E15 carrying pSR43.6#10 and pHZ-PHBcon	This study

Table S2. Plasmids used in this study

Plasmids	Description	Source
pHZ3.1(pSR58.6)	ColE1 ori, Cm^R, CcaR, *cpcG2* promoter, sfGFP	(Schmidl et al. 2014 [1])
pSR43.6	P15A ori, Sm^R, CcaS, *ho1*, *pcyA*	(Schmidl et al. 2014 [1])
pSR43.6#10	P15A ori, Sm^R, CcaS#10, *ho1*, *pcyA*	This study
pSR43.6#3	P15A ori, Sm^R, CcaS#3, *ho1*, *pcyA*	This study
pSR43.6#4	P15A ori, Sm^R, CcaS#4, *ho1*, *pcyA*	This study
pHZ-crRNA	pHZ3.1 containing crRNA	This study
43.6-crRNA	pSR43.6 containing crRNA	This study
pHZ-PHBcon	pHZ-crRNA plasmid containing constitutive *phbCAB* gene	This study
Paracr15A	P15A ori, Sm^R, araBAD operon and crRNA target *gltA* gene	Lab Stock
Paracr58.6	ColE1ori, Cm^R, araBAD operon and crRNA target *gltA* gene	This study

Table S3. Primers used in this study

Primer name	Sequence
pHZ.F	GATGGCCTTTTTGCGAAATACTAGATGCGTAAAGGCG
pHZ.R	CTCCTGCTAGCCCTCCTCTTTTTAAAAATGCGATCC
crR.F	CATTTTTAAAAAGAGGAGGGCTAGCAGGAGGAATTCAC
crR.R	TACGCATCTAGTATTTCGCAAAAAGGCCATCCGTCAG
phbCAB F2	ACAGAATCAGGGGGGGCAAGTACCTTGCCGACATCTATG
phbCAB R	CACATGTTCTTTCCTGCGTCTTCTGAATCCATGACCAG
phzzj F	GTCATGGATTCAGAAGACGCAGGAAAGAACATGTGAG
phzzj R	TCGGCAAGGTACTTGCCCCCCCTGATTCTGTGGATAAC
gRNAprimer F	GTAAAGGCGAAGAGCCATCGGTGATGTCGGCGATATAG
gRNAprimer R	TGAAGGCCTTTATCAGGTTATTGTCTCATGAGCGGATAC
58.6primer F	CATGAGACAATAACCTGATAAAGGCCTTCACATGGTCC
58.6primer R	CCGACATCACCGATGGCTCTTCGCCTTTACGCATTG
#3 F	GAATTATATGAGCAATTACAGCGACGCACGGAGGAAGTC

#3 R	CCGTGCGTCGCTGTAATTGCTCATATAATTCCGATTGTTG
#4 F	GGAATTATATGAGCAATTACAGCGCACGGAGGAAGTCCG
#4 R	CCTCCGTGCGCTGTAATTGCTCATATAATTCCGATTGTTG
#10F	TGAGCAATTACAGCTAGCTTTAGAACGGGAAAAAGAATTAAG
#10R	CGTTCTAAAGCTAGCTGTAATTGCTCATATAATTCCGATTGTTG
nocpc F	CAAAGCCCATTGTGCTTAAGGCGGTAATACGGTTATC
nocpc R	AACCGTATTACCGCCTTAAGCACAATGGGCTTTGCAG
ZJZL.FOR	AGGCCAGACTCCACCTGCAAAGCCCATTGTGCTTTTCTC
ZJZL.REV	TAGCGAGTCAGTGAGCGAGTCAGCGTCGTTACCAGAGTC
psr43.6#.FOR	GCACAATGGGCTTTGCAGGTGGAGTCTGGCCTCAAATAC
psr43.6#.REV	CTCTGGTAACGACGCTGACTCGCTCACTGACTCGCTAC

Table S4. Parameters of LED strips

LED strip					
LED type	3528 SMD(Surface Mount Device) LED				
LED number	60 LEDs/meter				
Circuit board width	6mm				
Nominal voltage	5V				
Waterproof	NO				
Characteristics	Soft LED strip.Each LED can be individually cut and plugged into the circuit.				
LED					
Basic specifications					
Size	Power		Operating temperature range		
3.5mm * 2.8mm * 1.9mm (Length * Width * Height)	0.06W		-40℃——80℃		
Welding temperature	Reverse voltage		Pulse current		
260℃	5V		100mA		
Product parameters					
Color	Working current(mA)	Voltage (V)	Luminous intensity (mcd)	Angle	The main wave band
Red	20mA	1.8-2.4	600-900	120°	620-630nm
Green	20mA	3.0-3.4	1000-1200	120°	520-530nm
Blue	20mA	3.0-3.4	300-500	120°	460-470nm

According to the international system of units , the definition of Radiant flux and the Definition of the Candela , the following formula for deriving the Radiant flux(Unit: W) of LEDs can be obtained:

$$\partial \Phi_e = \frac{I_V(\lambda) \times \partial \Omega}{683.002\, lm / W \times \bar{y}(\lambda)}$$

Φ_e : the radiant flux

$I_V(\lambda)$: the luminous intensity

Ω : the solid angle

$\bar{y}(\lambda)$: the photonic luminosity functions

∂ : the partial derivative symbol

Figure S1. The relative fluorescence of different type of CcaS-CcaR

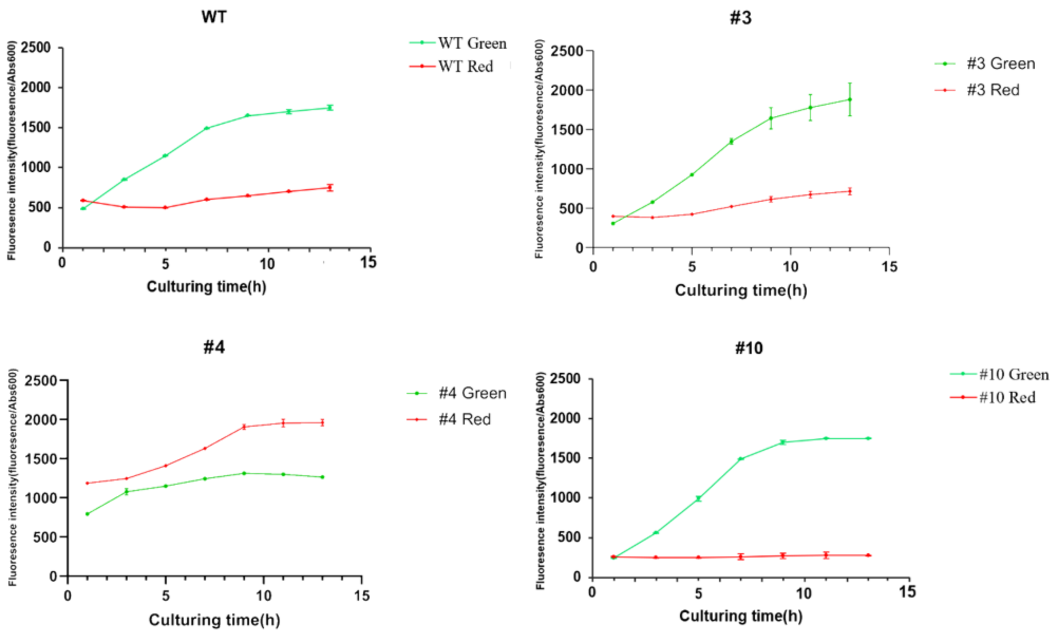

Figure S1. The relative fluorescence values of the cell cultures were measured under red and green light, respectively. Data represent the mean ± SD from three independent experiments (n = 3).

Figure S2. The modification of endogenous CRISPRi system

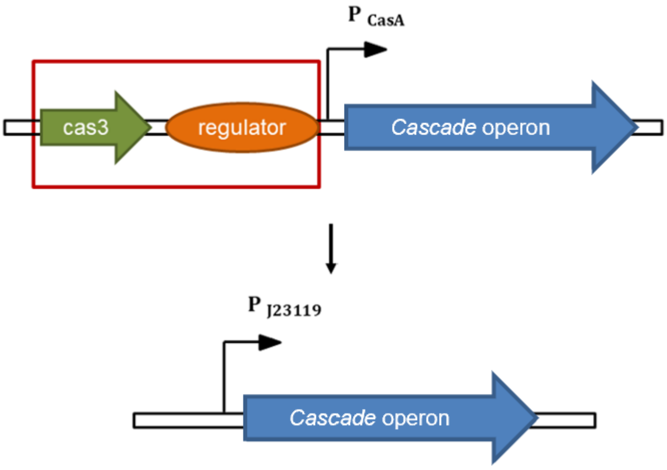

Figure S2 The construction of TOP10Δcas3. Cas3 is knocked out and promoter of Cascade is substituted by promoter J23119 [2].

Figure S3. sgRNA sequence targeting *gltA*

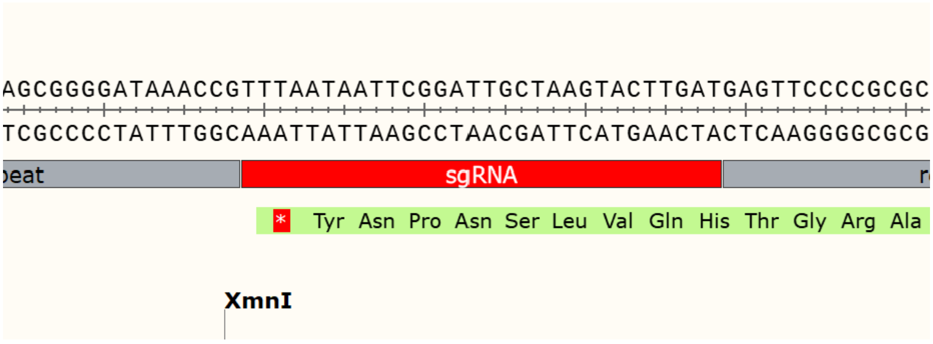

Figure S3. sgRNA sequence targeting *gltA*.

Figure S4. The regulation effect of different copy-number in light-controlled system

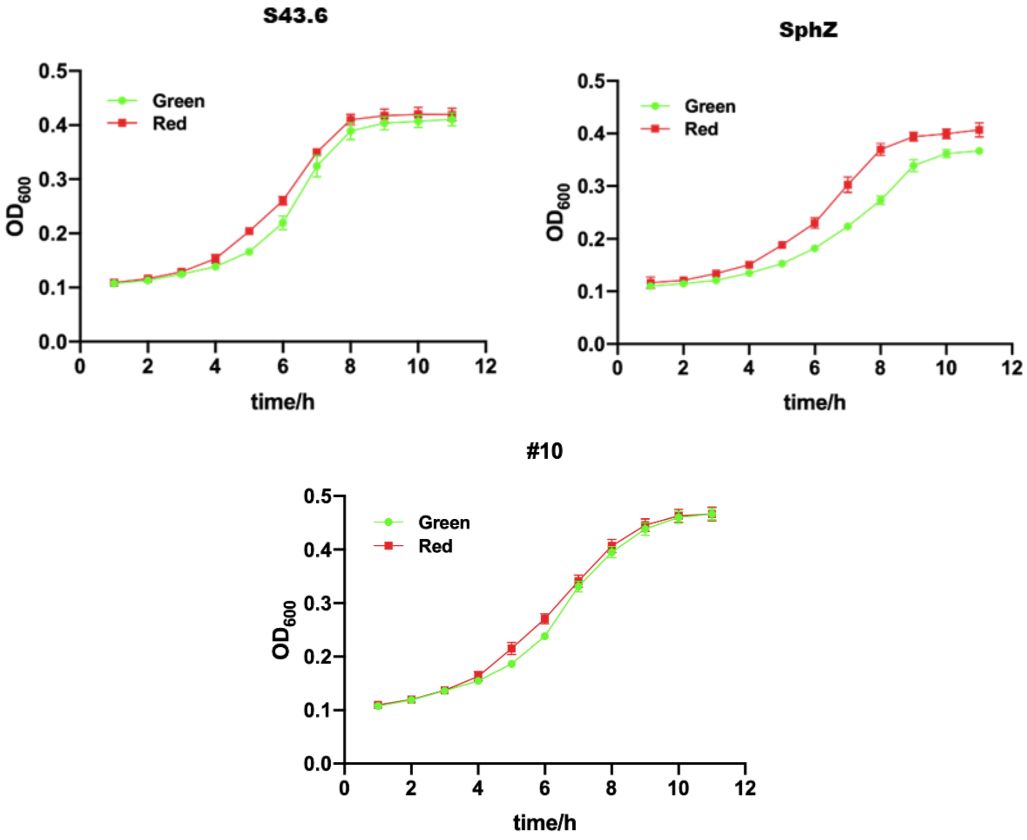

Figure S4. The regulation effect of different copy-number in light-controlled system. For S43.6, crRNA with tarting *gltA* spacer was constructed on medium-copy number plasmid (43.6-crRNA), while for strian SphZ, crRNA was on high-copy number plasmid(phZ-crRNA). Meanwhile #10 was negative control without crRNA. Data represents the mean ±SD from each of the three repeats(n=3).

References

1. Schmidl, S.R.; Sheth, R.U.; Wu, A.; Tabor, J.J. Refactoring and Optimization of Light-Switchable Escherichia coli Two-Component Systems. *ACS Synth. Biol.* **2014**, *3*, 820–831.
2. Chang, Y.; Su, T.; Qi, Q.; Liang, Q. Easy regulation of metabolic flux in Escherichia coli using an endogenous type I-E CRISPR-Cas system. *Microb. Cell Factories* **2016**, *15*, 195.
